# Bovine HEXIM1 inhibits bovine immunodeficiency virus replication through regulating BTat-mediated transactivation

**DOI:** 10.1186/1297-9716-44-21

**Published:** 2013-03-27

**Authors:** Hong-yan Guo, Yong-gang Ma, Yuan-ming Gai, Zhi-bin Liang, Jing Ma, Yang Su, Qi-cheng Zhang, Qi-min Chen, Juan Tan

**Affiliations:** 1 Key Laboratory of Molecular Microbiology and Biotechnology (Ministry of Education) and Key Laboratory Microbial Functional Genomics (Tianjin), College of Life Sciences, Nankai University, Tianjin, 300071, China; 2 College of Pharmacy and Tianjian Key Laboratory of Molecular Drug Research, Nankai University, Tianjin, 300071, China

## Abstract

The bovine immunodeficiency virus (BIV) transactivator (BTat) recruits the bovine cyclin T1 (B-cyclin T1) to the LTR to facilitate the transcription of BIV. Here, we demonstrate that bovine hexamethylene bisacetamide (HMBA)-induced protein 1 (BHEXIM1) inhibits BTat-mediated BIV LTR transcription. The results of in vivo and in vitro assays show direct binding of BHEXIM1 to the B-cyclin T1. These results suggest that the repression arises from BHEXIM1-BTat competition for B-cyclin T1, which allows BHEXIM1 to displace BTat from B-cyclin T1. Furthermore, we found that the C-terminal region and the centrally located region of BHEXIM1 are required for BHEXIM1 to associate with B-cyclin T1. Knockdown of BHEXIM1 enhances BIV replication. Taken together, our study provides the first clear evidence that BHEXIM1 is involved in BIV replication through regulating BTat-mediated transactivation.

## Introduction

Bovine immunodeficiency virus (BIV) belongs to the *Lentivirus* genus of the *Retroviridae* family. It was initially isolated from the leucocytes of an 8-year old Louisiana dairy cow, R-29, that had persistent lymphocytosis, lymphadenopathy, central nervous system lesions and wasting [1]. BIV infection has been detected in many countries around the world using serological or molecular assays [2-7]. Although most of these reports show BIV infection is asymptomatic, BIV seropositivity is variably associated with weight loss, secondary diseases, and diminished milk production [5,7,8].

The BIV genome, like human immunodeficiency virus (HIV) and other lentiviruses, contains the structural genes *gag*, *pol*, *env*, and several accessory genes, including *tat*, *rev*, *vif*, *vpw*, *vpy*, and *tmx*[9,10]. One of the most studied regulatory proteins is the Tat protein, which enhances viral gene expression [11]. The Tat proteins of different lentiviruses are divided into two groups depending on whether they bind TAR (transactivation response region) to elevate viral gene expression. The first group includes Tat proteins from BIV, Jembrana disease virus (JDV), HIV-1 and HIV-2, simian immunodeficiency virus (SIV), and equine infectious anemia virus (EIAV) [11,12]. These Tat proteins bind to the TAR present at the 5^′^ end of viral RNA transcripts and recruit the cellular positive transcription elongation factor b (P-TEFb), which consists of the cyclin-dependent kinase 9 (CDK9) and cyclin T1, to the viral promoter. Transcription elongation is enhanced through Tat-mediated recruitment and interaction with cyclin T1 and then CDK9-mediated phosphorylation of the carboxyl-terminal domain (CTD) of cellular RNA polymerase II (RNAP II) [13-19]. It is noteworthy that Tat from BIV (BTat) is distinct from other viruses of the first group because the recruitment of cyclin T1 does not contribute to TAR binding [12,20]. Tat proteins in the second group are from maedi-visna virus (MVV), caprine arthritis-encephalitis (CAEV) and feline immunodeficiency virus (FIV). These Tat proteins weakly transactivate their homologous LTR in a TAR-independent manner [21].

The hexamethylene bisacetamide (HMBA)-inducible protein 1 (HEXIM1) is a component of the inactive P-TEFb [22]. Upon binding to 7SK snRNA, HEXIM1 oligomers undergo a conformational change and then bind to cyclin T1. Following association with the P-TEFb complex, HEXIM1 inhibits the activity of CDK9 [23-26]. Through this mechanism, HEXIM1 suppresses HIV-1 Tat transactivation [27]. However, it is unknown whether HEXIM1 also interferes with BIV Tat transactivation. In this study, we show that bovine HEXIM1 (BHEXIM1) was associated with bovine cyclin T1 (B-cyclin T1) and suppressed BTat-mediated BIV LTR transcription activation. Our results further show that the nuclear localization signal and the C-terminal domain of BHEXIM1 were essential for binding to B-cyclin T1. Knockdown of BHEXIMI increased BIV replication, which further demonstrates the inhibitory effect of BHEXIM1 on BTat function.

## Materials and methods

### Materials

Protein A beads were purchased from Sigma (St. Louis, MO, USA). Anti-Flag M2 monoclonal antibody (MAb) was obtained from Stratagene (Agilent Technologies, Santa Clara, CA, USA). Anti-HA rabbit antibody, anti-HA mouse antibody, anti-β-actin C4 mouse antibody, horseradish peroxidase-conjugated anti-rabbit and anti-mouse secondary antibodies were obtained from Santa Cruz Biotechnology (Dallas, Texas, USA).

### Plasmids and proteins

pBIV-LTR-Luc and pHIV-LTR-Luc plasmids were constructed by subcloning BIV LTR or HIV LTR into a pGL3-basic luciferase reporter vector (Promega, Madison, WI, USA) upstream of the *luciferase* gene. The BIV LTR and HIV LTR were provided by Dr. Charles Wood (University of Nebraska Lincoln). pCMV-HA-BHEXIM1 and pCMV-HA-HHEXIM1 were generated by inserting BHEXIM1 or HHEXIM1 cDNA into the expression vector, pCMV-HA (Clontech, Mountain View, CA, USA). BHEXIM1 deletion mutants were created by PCR and cloned into pCMV-HA. The mammalian expression plasmid, Myc-BTat, was provided by Dr Matjaz Barboric (University of California). pcDNA3.1(+)-HTat (1-86 aa) was constructed by inserting HTat (1-86 aa) into the pcDNA3.1(+) vector (Invitrogen, CA, USA). The pcDNA-B-cyclin T1 (aa residues 1-272 of bovine cyclin T1) was a kind gift from Dr. Alan Frankel (University of California) [28] and was subcloned into the pCMV-Tag2B vector (Stratagene). The pGEX6p-1 (Amersham Bioscience, Pittsburgh, PA, USA) and pETH (Novagen, Darmstadt, Germany) vectors were used to construct bacterial expression plasmids for GST- and His-fusion proteins. *Escherichia coli* BL21 (DE3) strain cells were transformed with these constructs to express GST-B-cyclin T1, His-BHEXIM1, and His-BTat proteins. The proteins were purified with glutathione Sepharose 4B beads (GE healthcare, Pittsburgh, PA, USA) or His SepharoseTM 6 (GE healthcare) according to the respective manufacturer’s instructions.

### Cells and viruses

293 T, HeLa, fetal bovine lung (FBL) cells were primary cells isolated from fetal bovine lung by our laboratory; canine thymus cell line (Cf2Th), which is a BIV permissive cell line, was kindly provided by Prof. Jin-Ming Gao (Peking Union Medical College); and BIV baby hamster kidney indicator cells, BIVL, which harbor the BIV LTR-luciferase reporter [29], were maintained in Dulbecco’s Modified Eagle Medium (DMEM, Gibco, CA, USA) supplemented with 10% fetal bovine serum (FBS) and 2 mmol/L glutamine, 50 U/mL penicillin and 50 μg/mL streptomycin sulfate. All of the cells were cultured at 37°C in 5% CO_2_. The R29 strain of BIV was provided by Dr. Charles Wood (University of Nebraska Lincoln). Tissue culture infectious dose endpoint (TCID_50_) of BIV as BIV titers was evaluated by BIVL cells. Confluent BIVL cells on 96-well plates were infected with 25 μL of 10-fold serial dilutions of stock viruses with eight parallel wells for each dilution. Then the cells were incubated at 37°C in 5% CO2 for 48 h. Examination of luciferase activity was performed and the TCID_50_ was calculated with the Reed-Muench method [29].

### RNA isolation and RT-PCR

Total RNA was isolated from FBL cells using Trizol Reagent (Gibco) according to the manufacturer’s instructions. Fifteen micrograms of total RNA was used for cDNA synthesis using Moloney murine leukemia virus (M-MLV) reverse transcriptase (RT) (Promega). BHEXIM1 sequences were amplified by polymerase chain reaction (PCR) using the following primers: forward, 5^′^- GTGGAATTCAAATGGCCGAGCCACT-3^′^; reverse, 5^′^- AGCCTCGAGCTAGTCTCCAAAGTTG-3^′^.

### Small hairpin RNA (shRNA) treatments

The pSIREN-RetroQ (Clontech) was used to construct the shRNA-BHEXIM1 plasmid, which targets the CAGCGATGAGGACTTTATG sequence. A control shRNA targeting sequence GACAGAACCAGAGGATAGA, which does not pair with any eukaryotic mRNA, was used. The lentiviral shRNA system was used in this study as previously described [30]. Briefly, stock lentiviruses were produced by transfecting 293 T cells. Supernatants were harvested at 48 h post-transfection and were then stored at -80°C. To generate the BHEXIM1 downregulated cells, FBL cells were infected with stock lentiviruses for 12 h and then the virus was washed away. At 48 h after infection, puromycin (2 μg/mL) was added to the cultures to select for BHEXIM1-downregulated cells.

The amounts of BHEXIM1 and GAPDH mRNA in shRNA-BHEXIM1 or shRNA-control treated FBL cells were examined by real-time PCR. Real-time PCR was performed using the IQ5 Multicolor real-time PCR detection system (Bio-Rad, Hercules, CA, USA) and SYBR green real-time PCR master mix (Toyobo, Osaka, Japan) according to the manufacturers’ instructions. The sequences of the primer pairs were as follows: BHEXIM1 forward (5^′^-ACGACACCAGCGATGAGGAC-3^′^) and reverse (5^′^-TCCAGCCGCAGCCGATTATT-3^′^); BIV capsid protein-CA forward (5^′^-AAGGAGCCGTACACAGACTT-3^′^) and reverse (5^′^-TTCTGGAGCCGCCATACCTT-3^′^); and GAPDH, which was used as an internal control, forward (5^′^-AACGGCACAGTCAAGGCAGA-3^′^) and reverse (5^′^-TCGGCAGAAGGTGCAGAGAT-3^′^). The specificity of the amplification reaction was verified by melting-curve analyses. Threshold cycle (*C*_*T*_) values were calculated automatically as the cycle when the sample fluorescence exceeded a threshold level corresponding to 10 standard deviations (SD) of the mean of the baseline fluorescence. The level of BHEXIM1 and CA expression was normalized to GAPDH using the 2^-ΔΔ*CT*^ method [31].

### Luciferase assay

Cf2Th cells were plated in 12-well plates (1 × 10^5^/well) and cultured at 37°C for 20 h before transfection using polyethylenimine (PEI; sigma). The total DNA in each transfection mixture was normalized using empty vector DNA. pCMV-β-gal plasmid DNA (0.1 μg) was included in each transfection. Cells were harvested at 48 h after transfection. The luciferase activity was measured using a commercial assay system (Promega). The cells were rinsed with PBS and lysed with lysis buffer (Promega). The cell lysates were then clarified from the insoluble materials by centrifugation at 12 000 rpm for 3 min. The clarified lysate (5 μL) was mixed with the luciferin reagent (50 μL) and luciferase activity was measured in a chemiluminescence measurement machine (Promega). Simultaneously, 50 μL clarified lysate was mixed with the same volume (50 μL) of o-Nitrophenyl-β-D-Galactopyranoside (ONPG) buffer (4.4 mM ONPG, 4.3 mM MgCl_2_-6H_2_O, 164 mM Na_2_HPO_4_-12H_2_O, 36 mM NaH_2_PO_4_-2H_2_O, 0.684% β-mercaptoethanol). The activity of β-galactosidase was then examined at OD_405_ nm with a Precision Microplate Reader (Molecular Devices, Sunnyvale, CA, USA) until the results ranged between 0.200 and 0.800. The results were used as an internal control to normalize the efficiency between transfections by the formula: (each well of luciferase activity) / (each well of β-galactosidase activity - the background of β-galactosidase activity). The luciferase activity of each well divided by the control well was expressed as the relative luciferase activity (fold). Each experiment was repeated at least three times.

### Western blotting

Cf2Th cells plated in 12-well plates (1 × 10^5^/well) were transfected with various plasmids. Fourty-eight hours post-transfection, the cells were harvested and washed twice with 1× phosphate buffered saline. The cells were then lysed with NP-40 lysis buffer (0.5% NP-40, 0.1% Triton X-100, 0.1% sodium deoxycholate, 10 mM Tris-Cl, 150 mM NaCl, 1 mM EDTA) containing 1% PMSF for 30 min on ice. After centrifugation at 10 000 × *g* for 10 min at 4°C, the supernatants were collected, and protein concentrations were measured using the Bio-Rad Protein Assay reagent (Catalog No. #500-0006). The samples (30 μg/lane) were separated by 12% polyacrylamide gel electrophoresis (SDS-PAGE). The proteins were then transferred onto a polyvinylidene difluoride (PVDF) membrane (Millipore, Billerica, MA, USA) by electroblotting for 1 h at 100 V, 4°C. Following incubation in 5% nonfat milk (in 1× phosphate-buffered saline) for 45 min at room temperature, the membrane was blotted with primary antibody for 90 min at room temperature and then incubated with goat anti-mouse or goat anti-rabbit secondary antibodies conjugated with peroxidase.

### Co-immunoprecipitation (Co-IP) and Pulldown assays

For the co-immunoprecipitation assay, a total of 1 × 10^7^ 293 T cells in 100-mm-diameter dishes were transfected with various plasmids using the PEI reagent. Cells were harvested at 48 h after transfection; lysed in 600 μL lysis buffer (50 mM Tris-HCl [pH8.0], 150 mM NaCl, 1% NP-40, and 1 mM phenylmethylsulfonyl fluoride); sonicated; and centrifuged for 15 min at 4°C, 10 000 × *g*. The supernatant was incubated with antibody at 4°C for 2 h. Thirty microliters of protein A beads was then added, and the mixture was incubated for an additional 2 h at 4°C. Immunocomplexes were washed five times with the lysis buffer and then boiled in SDS loading buffer. The precipitated proteins were detected by SDS-PAGE and immunoblotting. For the GST pulldown assay, purified GST and GST-fusion proteins were immobilized on glutathione Sepharose 4B beads, and then incubated with purified His-fusion proteins. After extensive washing, the beads were boiled in SDS loading buffer and the proteins were subjected to SDS-PAGE and immunoblotting.

## Results

### BHEXIM1 inhibits LTR transcriptional activation by BTat

**Figure 1:**
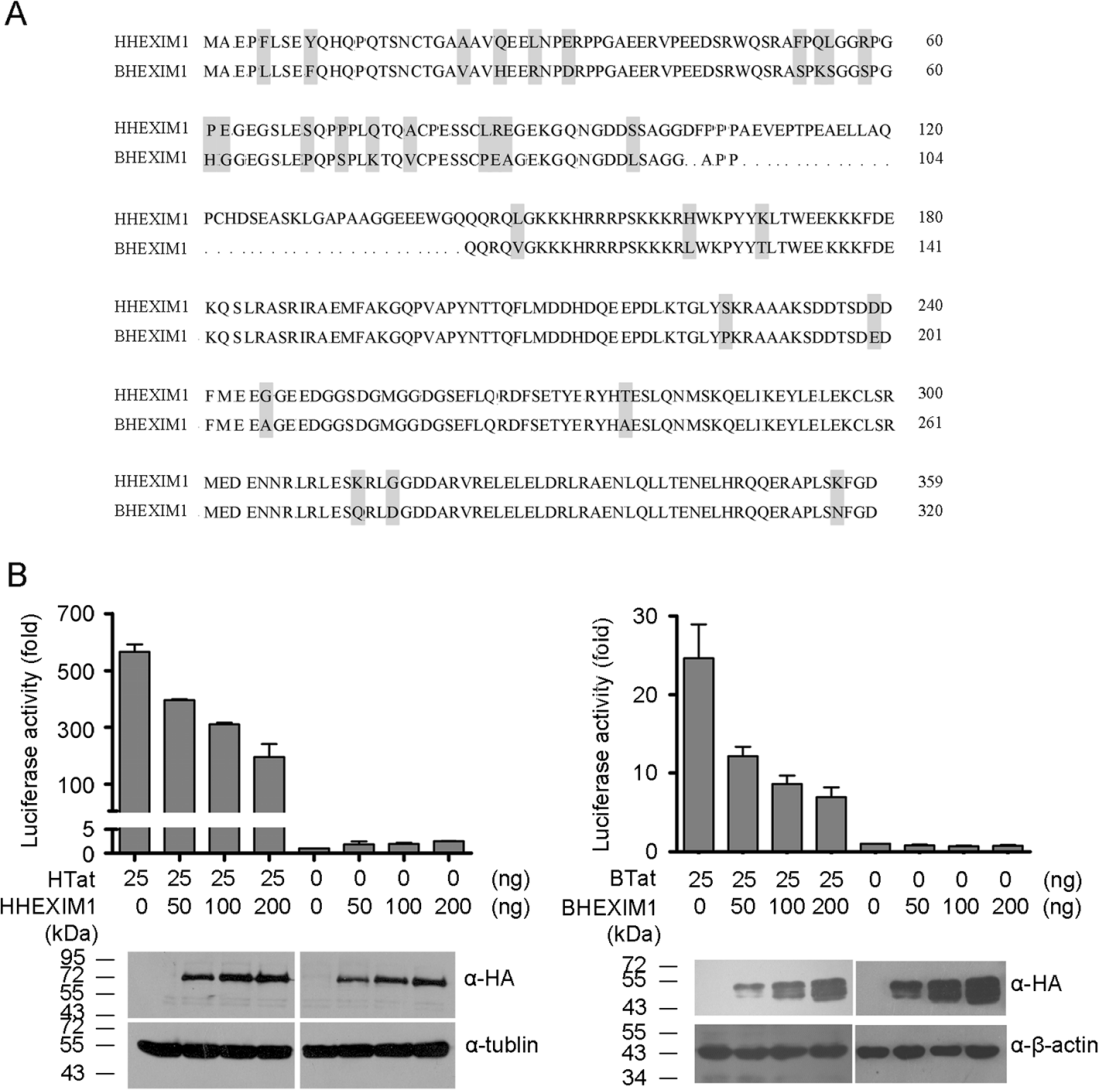
**BHEXIM1 inhibits BTat transactivation of LTR transcription.** (**A**) Sequence comparison of the bovine and human HEXIM1 proteins. Amino acid differences between the two proteins are shown in shaded boxes. Amino acid deletion (•) is indicated. (**B**) HeLa cells were transfected with 0.05 μg of the reporter plasmid pHIV LTR-Luc, 0.01 μg of the pCMV-β-gal plasmid, and combined with the indicated amount of HA-HHEXIM1 and with or without HTat (0.025 μg) (left panel). Cf2Th cells were co-transfected with the BIV LTR-Luc plasmid (0.05 μg), the pCMV-β-gal plasmid (0.1 μg), and the indicated amount of the HA-BHEXIM1 expression plasmid with or without BTat (0.025 μg) (right panel). After 48 h, the relative luciferase activities were analyzed. The relative expression of HA-HHEXIM1 or HA-BHEXIM1 in the transfected cells was monitored by WB assay (bottom panel). Error bars represent the SD of the means from three independent experiments.

We first cloned the BHEXIM1 gene [GenBank: NM_001076181] from FBL cells by RT-PCR. BHEXIM1 has 320 amino acids and is therefore 39 amino acids shorter than its human counterpart. The amino acid residues 102, 103 and 107 to 143 in human HEXIM1 are missing in BHEXIM1. The human and bovine HEXIM1 mainly differ in their N-terminal regions (Figure 1A). We first examined the function of HHEXIM1 on HTat transactivation. HeLa cells were transfected with a luciferase reporter plasmid under the control of the HIV LTR, along with increasing amounts of HHEXIM1 with or without HTat. The results show that HHEXIM1 reduced HTat-mediated HIV LTR transactivation in a dose-dependent manner (Figure 1B, left panel). We next asked whether BHEXIM1 modulates the function of BTat in transactivating the BIV LTR. To answer this question, the BIV-LTR-Luc reporter construct was used to monitor the transcriptional activity of the BIV LTR promoter. A BIV permissive cell line, Cf2Th, was transfected with pLTR-Luc reporter plasmid and increasing amounts of BHEXIM1 in the absence or presence of BTat. As shown in Figure 1B (right panel), BHEXIM1 inhibited BTat-induced transactivation in a dose-dependent manner. Therefore, the results suggested that BHEXIM1 diminishes the transactivation of the BIV LTR by BTat.

### BHEXIM1 interacts directly with B-cyclin T1

**Figure 2:**
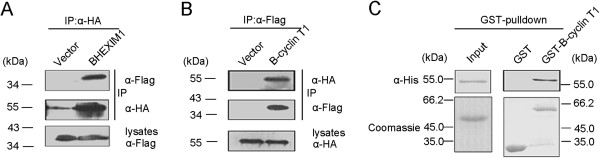
**BHEXIM1 interacts directly with B-cyclin T1.** (**A**) Co-IP of BHEXIM1 and B-cyclin T1. 293 T cells were co-transfected with Flag-B-cyclin T1 and either an empty vector (lane 1) or HA-BHEXIM1 (lane 2). BHEXIM1 was immunoprecipitated with rabbit anti-HA agarose beads, and the immunoprecipitates were detected by western blot using mouse anti-Flag (top) or mouse anti-HA (middle) antibodies. The amount of B-cyclin T1 in each cell lysate was checked using a mouse anti-Flag antibody (bottom). (**B**) Reciprocal Co-IP of BHEXIM1 and B-cyclin T1. 293 T cells were co-transfected with HA-BHEXIM1 and either an empty vector (lane 1) or Flag-B-cyclin T1 (lane 2). B-cyclin T1 was immunoprecipitated with mouse anti-Flag agarose beads, and the immunoprecipitates were detected by western blot using rabbit anti-HA (top) or mouse anti-Flag (middle) antibodies. The amount of BHEXIM1 in the cell lysates was checked using a rabbit anti-HA antibody (bottom). (**C**) In vitro interaction between BHEXIM1 and B-cyclin T1. The purified BHEXIM1 protein was incubated with GST or GST-B-cyclin T1 agarose beads. GST pulldown was then performed, and precipitates were detected by western blot using an anti-His antibody. Purified His-BHEXIM1, GST and GST-B-cyclin T1 used in the pulldown assay were detected by Coomassie blue staining.

To determine whether BHEXIM1 associated with P-TEFb, we first tested the interaction between BHEXIM1 and B-cyclin T1. 293 T cells were co-transfected with HA-BHEXIM1 and Flag-B-cyclin T1 constructs. Following immunoprecipitation with HA antibodies, the precipitates were examined by western blot with anti-Flag antibodies. The results show that Flag-B-CyclinT1 was co-immucx cnoprecipitated with HA-BHEXIM1 (Figure 2A). Similarly, HA-BHEXIM1 was co-immunoprecipitated with Flag-B-cyclin T1 (Figure 2B). To determine whether BHEXIM1 interacted directly with B-cyclin T1 in vitro, His-BHEXIM1 and GST-B-cyclin T1 were expressed in *E.coli* and purified using the protein expression system. The results of the GST pulldown assay show that His-BHEXIM1 was eluted with GST-B-cyclin T1, suggesting a direct association between BHEXIM1 and B-cyclin T1 (Figure 2C). Taken together, these results demonstrate a direct interaction between BHEXIM1 and B-cyclin T1.

### BHEXIM1 interferes with the interaction of BTat and B-cyclinT1

**Figure 3:**
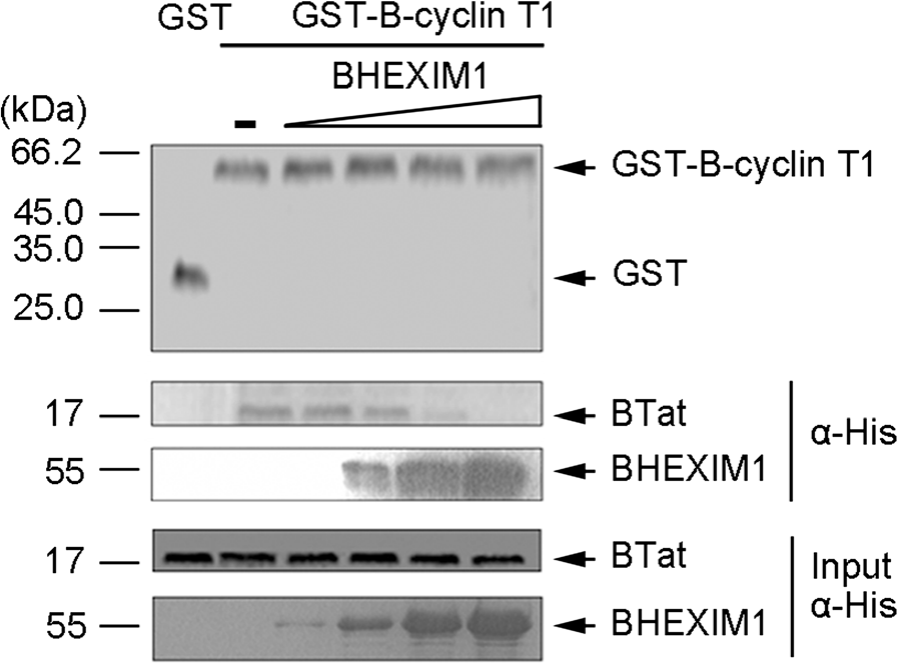
**BHEXIM1 competes with BTat for binding B-cyclin T1.** GST (lane 1, 20 μg) or GST-B-cyclin T1 (lane 2-6, 20 μg) was incubated with His-BTat (5 μg). Increasing amounts of His-BHEXIM1 (1, 5, 10, 20 μg) were added to the reactions in lane 3-6 but not lane 1 and 2. GST pulldown was then performed, and the purified GST and GST-B-cyclin T1 used in the pulldown assay were detected by Coomassie blue staining (top). The bound BTat and BHEXIM1 were detected using an anti-His antibody (middle). The amount of BTat and BHEXIM1 in the cell lysates were checked using an anti-His antibody (bottom).

Previous studies demonstrated that Tat stimulates the synthesis of viral mRNA via the interaction with cyclin T1 [32] and showed that HHEXIM1 and HIV-1 Tat binds cyclin T1 in a mutually exclusive fashion [33,34]. To test whether BHEXIM1 competed with BTat for binding to B-cyclin T1, we used purified recombinant proteins to study the interactions of BHEXIM1 and BTat with GST-B-cyclin T1 (aa 1-272). The data in Figure 3 show that BTat bound to GST-B-cyclin T1 but not GST alone in the absence of BHEXIM1 (compare lanes 1 and 2). However, BHEXIM1 protein reduced the amount of BTat bound to GST-B-cyclin T1 (compare lanes 4-6 to 2). When the ratio of BHEXIM1 and BTat reached 4:1 (lane 6), BTat was completely displaced from GST-B-cyclin T1 by BHEXIM1. These results suggest that BHEXIM1 competes with BTat in binding to B-cyclin T1.

### Identification of protein sequences involved in BHEXIM1 binding to B-cyclin T1 and inhibiting the transactivation function of BTat

**Figure 4:**
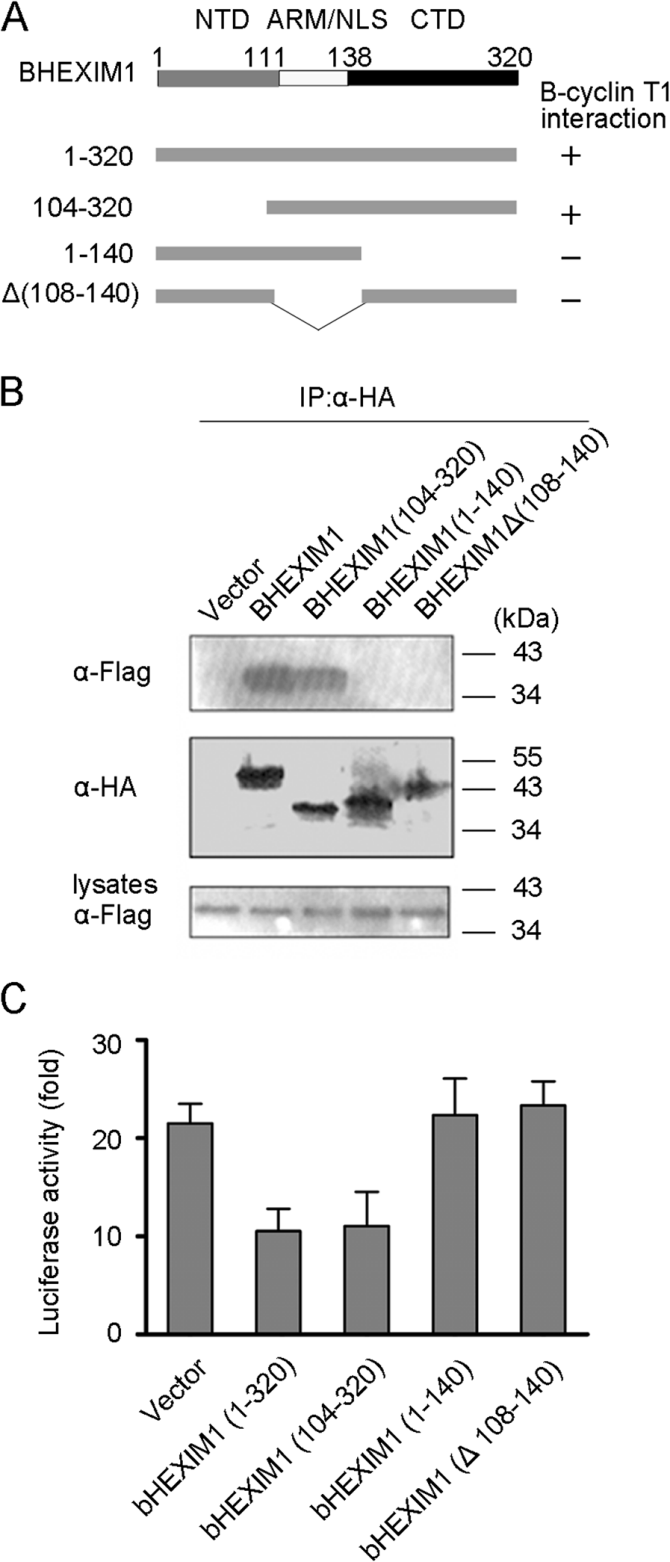
**Identification of the BHEXIM1 domain responsible for binding to B-cyclin T1 and inhibiting BTat transactivation.** (**A**) Schematic representation of HA-tagged truncation forms of BHEXIM1. (**B**) 293 T cells were co-transfected with an empty vector, BHEXIM1, or the indicated truncations together with Flag-B-cyclin T1. BHEXIM1 was immunoprecipitated with rabbit anti-HA agarose beads, and the immunoprecipitates were detected by western blot using a mouse anti-Flag (top) or mouse anti-HA (middle) antibody. The amount of B-cyclin T1 in the cell lysates was checked using an anti-Flag antibody (bottom). (**C**) Cf2Th cells were co-transfected with a BIV LTR-Luc plasmid (0.05 μg), pCMV-β-gal plasmid (0.1 μg), wild-type BHEXIM1 or the deletion form of BHEXIM1 (0.1 μg) with or without BTat (0.025 μg), and an empty vector to ensure that the total DNA concentration in each transfection mixture was constant. After 48 h, the relative luciferase activities were analyzed. All of the luciferase activities with Tat were divided by the luciferase activities without Tat. Error bars represent the SD of the means from three independent experiments.

We next investigated which regions of BHEXIM1 are required for binding to B-cyclin T1. To this end, we generated three BHEXIMI mutants named 104-320, 1-140 and Δ(108-140) that lack the amino acids 1-104, 140-320 and 108-140 respectively (Figure 4A). 293 T cells were then transfected with plasmids expressing either the wild-type or truncated BHEXIM1 tagged with HA, together with the Flag-B-cyclin T1 plasmid. A co-immunoprecipitation assay shows that 1-320 aa (full-length) and 104-320 mutant were able to interact with Flag-B-cyclin T1, but the 1-140 and Δ(108-140) mutant lost the interaction (Figure 4B). Therefore, the central region between residues 108 and 140 and the C-terminal domain of BHEXIM1 were essential for binding to B-cyclin T1. Cf2Th cells were also transfected with the LTR-Luc reporter plasmid and different truncations of BHEXIM1, with or without BTat. The results show that the removal of the N-terminal amino acids of BHEXIM1 (104-320) did not eliminate the inhibition. However removal of the C-terminal amino acids (1-140) and the central region significantly affected the inhibition (Figure 4C). Thus, BHEXIM1-mediated repression required the presence of the C-terminal domain and the central region between residues 108 and 140.

### Knockdown of BHEXIM1 enhances BIV replication

**Figure 5:**
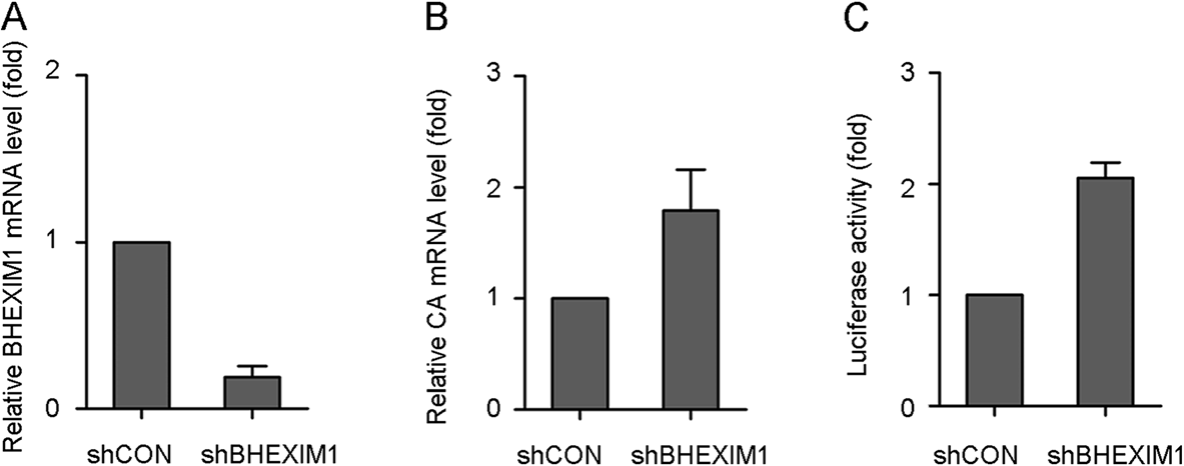
**RNAi Knockdown of BHEXIM1 in FBL cells enhances BIV replication.** (**A**) FBL cells were stably transfected with either BHEXIM1-specific (shBHEXIM1) or control (shCON) shRNA oligonuceotides. Cellular RNA was extracted and subjected to real-time PCR to examine the amount of BHEXIM1 and GAPDH. (**B**) BHEXIM1-knockdown (shBHEXIM1) and control (shCON) FBL cells (5 × 10^5^/well) were infected with 5 × 10^3^ TCID_50_ BIV. The mRNA level of BIV CA was examined by real-time PCR at 24 h post-infection. (**C**) BHEXIM1-knockdown (shBHEXIM1) and control (shCON) FBL cells (5 × 10^4^/well) were infected with 5 × 10^2^ TCID_50_ BIV. At 24 h post-infection, BIVL (1 × 10^5^/well) was added and the luciferase activity was assayed after 48 h co-culture. Error bars represent the SD of the means from three independent experiments.

We next measured the effect of knockdown of endogenous BHEXIMI on BIV infection. First, we transduced the FBL cells with shRNA targeting BHEXIMI and generated a stable knockdown cell line with an 80% decrease of BHEXIM1 mRNA (Figure 5A). We then used BIV to infect the BHEXIM1 knockdown cells and control cells. Twenty-four hours after infection, the levels of viral RNA were measured by RT-PCR. As shown in Figure 5B, the viral RNA level was increased by approximately 2-fold in BHEXIM1 stable-knockdown cells compared to control cells. To determine the amounts of infectious BIV produced, the infected BHEXIM1 knockdown FBL cells or control cells were co-cultured with the BIV indicator cell line, BIVL, and the luciferase activity was measured 48 h later. The results show an approximately two-fold increase in luciferase activity (Figure 5C). Taken together, the data suggest that BIV replication was enhanced when BHEXIM1 was down-regulated in infected cells.

## Discussion

Viruses are obligate intracellular parasites and must employ cellular machinery for their efficient replication. Meanwhile, host cells evolve different strategies to suppress or eliminate viral infection and replication. BTat is an early regulatory protein, and it plays an essential role in the BIV life cycle. Transcriptional transactivation of the BIV LTR by essential BTat requires recruitment of cyclin T1 to the BIV TAR to enhance viral transcription elongation. HEXIM1 as a host cellular P-TEFb inhibitor may regulate Tat function and viral replication.

Our study was the first one to report the role of bovine HEXIM1 in BTat transactivation and BIV replication. Our data demonstrate that BHEXIM1 inhibited BTat-mediated BIV LTR transactivation by direct interaction with B-cyclin T1. The results of the competition assay further show that BHEXIM1 disrupted BTat binding to B-cyclin T1. Finally, knockdown of BHEXIM1 increased BIV gene expression and production of BIV, indicating the regulatory role of BHEXIMI involved in BIV infection.

BHEXIM1 and HHEXIM1 share 80.50% homology in their amino acid sequences and both proteins have three main domains. Comparing to other parts of the two proteins, the N-terminal domain (NTD) exhibited a lower homology (57.05%), which is reported to be the self-inhibitory domain [35], because a 39 amino acid deletion was found in BHEXIM1. The central region contains an arginine-rich motif (ARM) and functions as the nuclear localization signal (NLS). This region serves as the 7SK-binding site [26,36] and shows 92.86% homology between HHEXIM1 and BHEXIM1. Only two amino acids differ in the central regions of BHEXIM1 and HHEXIM1. The C-terminal domain is rich in acidic residues and shows 96.17% homology between HHEXIM1 and BHEXIM1. This region contains the coiled-coil domain and the conserved PYNT motif. In HHEXIM1, this region mediates direct interaction with P-TEFb [26]. The high homology between BHEXIM1 and HHEXIM1, especially in their central and C-terminal regions, indicates their conserved structures and functions.

A recent report suggested that HTat competes with HHEXIM1 for binding to cyclin T1 [33], displaying the reciprocal competition between HHEXIM1 and cyclin T1. The apparent discrepancy may be explained by the fact that higher amounts of BHEXIM1 compared to BTat exhibited the advantage of binding B-cyclin T1 and could displace BTat from B-cyclin T1. The central region of BHEXIM1, which may be involved in nucleus localization and 7SK RNA binding, was also proved to be important in BHEXIM1 binding B-cyclin T1 and inhibiting the transactivation function of BTat, consistent with previously demonstrated roles of HHEXIM1 on HIV [26,36]. When the native HEXIM1 NLS was replaced with a foreign NLS sequence, the NLS-substituted HEXIM1 mutants failed to interact with P-TEFb. Thus, the arginine-rich 7SK-binding motif within the NLS of HEXIM1 was indispensable for HEXIM1’s inhibitory effect on transcription through 7SK as a molecular scaffold-mediated interaction with P-TEFb. The essential role of this domain indicated the crucial function of 7SK RNA in the interaction between BHEXIM1 and B-cyclin T1 and the potential role of BHEXIM1/7SK RNA interaction in BTat inhibition. Future studies on the function of 7SK RNA in BIV transactivation may be necessary to evaluate the proposed hypothesis.

In summary, this study demonstrates the repression of BHEXIM1 on BTat transactivation by competing with BTat for binding to B-cyclin T1, allowing it to directly displace BTat from B-cyclin T1. Furthermore, we found that the C-terminal region and the centrally located 7SK snRNA recognition region of BHEXIM1 are essential for repression. Knockdown of BHEXIM1 enhances BIV replication. Based on the data presented in this study, we propose the potential impact of BHEXIM1 on the BIV latent life cycle and the absent clinical disease signs of infected livestock. This finding may provide important information for BIV molecular biology and clinical diagnoses and prevention.

## Competing interests

The authors declare that they have no competing interests.

## Authors’ contributions

HYG, YS, and JT conceived and designed the experiments. HYG, ZBL, YMG, and JM performed the experiments. HYG, YGM, and JT analyzed the data. QCZ and QMC contributed reagents/materials/analysis tools. HYG and JT composed the manuscript. All of the authors read and approved the final manuscript.
